# Preliminary evaluation of SaCoVLM™ video laryngeal mask airway in airway management for general anesthesia

**DOI:** 10.1186/s12871-021-01541-0

**Published:** 2022-01-03

**Authors:** Chun-ling Yan, Ying Chen, Pei Sun, Zong-yang Qv, Ming-zhang Zuo

**Affiliations:** 1 Department of Anesthesia, Beijing Hospital, National Center of Gerontology, Institute of Geriatric Medicine, Chinese Academy of Medical Sciences, Beijing, PR China; 2 Peking University First Hospital, Xishiku Street, Xicheng District, Beijing, PR China

**Keywords:** Airway management, Glottic exposure classification, General anesthesia, SaCoVLM™ video laryngeal mask, Sealing pressure

## Abstract

**Background:**

To preliminary evaluate the application of SaCoVLM™ video laryngeal mask airway in airway management of general anesthesia.

**Methods:**

We recruited 100 adult patients (ages 18–78 years, male 19, female 81, weight 48–90 kg) with normal predicted airway (Mallampati I ~ II, unrestricted mouth opening, normal head and neck mobility) and ASA I-II who required general anaesthesia. The SaCoVLM™ was inserted after anesthesia induction and connected with the anesthesia machine for ventilation. Our primary outcome was glottic visualization grades. Secondary outcomes included seal pressure, success rate of insertion, intraoperative findings (gastric reflux and contraposition), gastric drainage and 24-h complications after operation.

**Results:**

The laryngeal inlet was exposed in all the patients and shown on the video after SaCoVLM™ insertion. The status of glottic visualization was classified: grade 1 in 55 cases, grade 2 in 23 cases, grade 3 in 14 cases and grade 4 in 8 cases. The first-time success rate of SaCoVLM™ insertion was 95% (95% CI = 0.887 to 0.984), and the total success rate was 96% (95% CI = 0.901 to 0.989). The sealing pressure of SaCoVLM™ was 34.1 ± 6.2 cmH_2_O and the gastric drainage were smooth. Only a small number of patients developed mild complications after SaCoVLM™ was removed (such as blood stains on SaCoVLM™ and sore throat).

**Conclusions:**

The SaCoVLM™ can visualize partial or whole laryngeal inlets during the surgery, with a high success rate, a high sealing pressure and smooth gastroesophageal drainage. SaCoVLM™ could be a promise new effective supraglottic device to airway management during general anesthesia.

**Trial registration:**

ChiCTR,ChiCTR2000028802.Registered 4 January 2020.

## Background

Since the introduction of laryngeal mask in the late 1980s, the “blind” insertion technique described by Dr. Brain has been widely used in clinical practice [1]. Previous studies found that approximately 40 to 60% of the laryngeal masks inserted blindly did not achieve ideal alignment with bronchofiberscope, and some even require re-alignment to improve ventilation [2, 3]. In realigning the laryngeal mask, complications, such as hypoxia and laryngeal spasm, might occur [4]. Technical innovation should be made to assure the perfect alignment of laryngeal mask and minimize adverse airway events. SaCoVLM™ video laryngeal mask (SaCoVLM™ ZHEJIANG UE MEDICAL CORP. Add:No.8, Youyi Road, Baita Economic Develop Zone, Xianju, Zhejiang, China) has been recently invented. Using new technology combining camera and Laryngeal mask, it can visualize the conditions around the glottis, thus achieving rapid and accurate insertion. In addition, during the maintenance of anesthesia, the conditions around the glottis could be monitored, through which the position of SaCoVLM™ could be corrected in time to prevent aspiration of perilaryngeal secretions and reduce stimuli to the throat. The purpose of this study was to observe the visibility of SaCoVLM™ video laryngeal mask in clinical use and to preliminarily evaluate its application in airway management under general anesthesia.

## Methods

### Subjects

This single-center prospective observational study was approved by the Ethics Committee of Clinical Research of Beijing Hospital (No. 2019BJYYEC-236-02) and registered in the China Clinical Trial Registration Center (No. ChiCTR2000028802 Date2020.01.04). Between February 2020 and December 2020, 100 adult patients who received SaCoVLM™ for general anesthesia were recruited and informed consent was obtained. The sample size of 100 cases is based on a similar preliminary evaluation of a new laryngeal mask airway by Liu et al. [5]. Inclusion criteria: age ≥ 18; gender unrestricted; ASA I-II; 18 kg/m^2^ ≤ BMI ≤ 30 kg/m^2^; Normal airway. Exclusion criteria: severe respiratory diseases; lateral or prone surgical positions; mouth opening less than 2 cm; edentulous; the presence of risk factors for gastric reflux or aspiration, including fasting, morbid obesity, gestation over 14 weeks, ileus, and hiatal hernia; other laryngeal mask contraindications, included intraoral, laryngeal surgery and thoracic surgery.

All the methods in this study were performed in accordance with the relevant guidelines and regulations in the Methods section.

### Preparation of SaCoVLM™

**Fig. 1:**
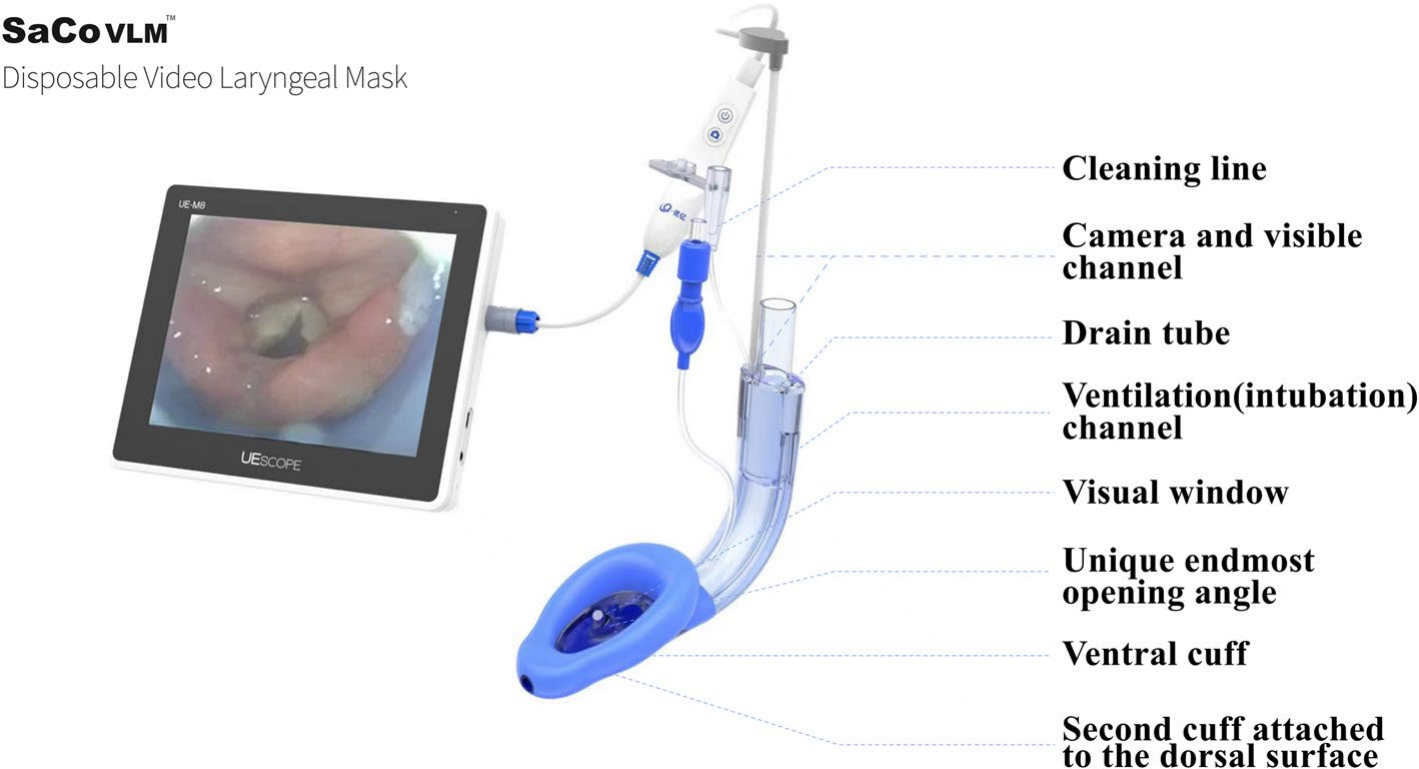
SaCoVLM™ disposable video laryngeal mask

Disposable SaCoVLM™ (Fig. 1) includes a visual channel, a ventilation (intubation) channel, a gastric tube channel, a camera (electronic camera, focal length 7 mm, field angle 90° ± 13.5%, ZHEJIANG UE MEDICAL CORP.) and connecting wires. The camera is fixed on the right side of ventral cuff, connected with the screen and inserted into the visual channel. During placement, the SaCoVLM™ is adjusted according to the image displayed on the screen. The data are stored in a chip. A rechargeable battery is used to provide energy. The SaCoVLM™ was selected according to the patient’s weight. Size 3 was used for patients weighing 30–50 kg, size 4 for patients weighing 50–70 kg, and size 5 for patients weighing 70–90 kg. Before placement, the cuff was deflated and flattened. The back of the laryngeal mask was lubricated with Lidocaine Hydrochloride Gel. The camera was inserted into the visual channel and connected with the screen before later use.

### Preoperative preparation

All subjects were forbidden to drink for 6 h and fasted for 8 h before operation. The general information of the patients was asked before surgery, including age, height and weight. We measured the thyromental distance, the mouth opening of the patients, Mallampati classification, upper lip occlusion test and ASA classification**.**

Peripheral venous access was initiated in the operating room. Electrocardiogram (ECG), heart rate (HR), blood pressure (BP), oxygen saturation (SpO_2_) and bispectral index (BIS) were monitored.

### Anesthesia and airway management

The patient was preoxygenated with 100% oxygen (5 L/min, 5 min) before induction using a facemask and the head was placed in the neutral supine position. General anesthesia was induced with sufentanil (0.2–0.5 μg/kg), propofol (2 mg/kg) and cis-atracurium (0.2 mg/kg). The patient’s lungs were manually ventilated when the BIS was reduced to below 60. After obtaining easy mask ventilation and a relaxed airway, the SaCoVLM™ was inserted. The anesthetist held the distal end of the ventilation channel and let the laryngeal mask slide down the palatopharyngeal curve along midline in the mouth, until the front end of SaCoVLM™ was inserted into the hypopharyngeal cavity. The SaCoVLM™ was inflated to achieve a maximum cuff pressure of 60 cmH_2_O (1 cmH_2_O = 0.098 kpa) detected by a hand-held manometer (VBM, German). Artificial ventilation was performed. The glottic visualizaiton was observed and a gastric tube (12Fr) was placed through the gastroesophageal channel. After proper fixation of SaCoVLM™, positive pressure ventilation was performed. Mechanical ventilation parameters were set: tidal volume 6–8 mL/kg, inhalation/exhalation ratio 1:2, ventilation frequency 12 times/min and P_ET_CO_2_ 35–45 mmHg (1 mmHg = 0.133 kPa). Fibreoptic bronchoscopy was performed to grade the glottic exposure. The SaCoVLM™ insertion was considered successful when the following criteria were met: chest movement and no air leakage during normal ventilation; more than two continuous end-expiratory carbon dioxide waveforms; positive suprasternal concave gel test; the gastric tube successfully implanted and the ventilation channel connected to the anesthesia machine for mechanical ventilation. The criteria of SaCoVLM™ insertion failure: failure to place the SaCoVLM™ correctly after more than 2 times and longer than 60 s; abnormal P_ET_CO_2_ waveforms; bellows collapsing. All procedures were performed by the same anesthesiologist experienced in the use of SaCoVLM™. Time of SaCoVLM™ insertion referred to the time between SaCoVLM™-incisors contact and the first ventilation showing two P_ET_CO_2_ waveforms. If the air leakage was obvious, the position of SaCoVLM™ needed to be adjusted using one or more of the following methods: the up-down maneuver, raising the jaw with both hands, the Chandy maneuver, increasing and reducing the amount of air, re-insertion and changing the size. The position and function of SaCoVLM™ was reevaluated after adjustment. If the insertion failed twice, endotracheal intubation was performed.

The pressure gauge stability method was used to measure the sealing pressure inside the SaCoVLM™ [6]. The fresh gas flow rate was adjusted to 3 L/min, the pressure was adjusted to 40 cmH_2_O, and mechanical ventilation was switched to manual ventilation. When the sound of air leakage was heard, the pressure was gauged as the sealing pressure. For the sake of safety, the maximum oropharyngeal leak pressure was set to be 40 cmH_2_O.

Anesthesia was maintained with targeted-infusion of propofol (2.5–3.5 μg/ml) and remifentanil (3-4 ng/ml), punctuated with infusion of cisatracurium. BIS was controlled at 40–60. Propofol and remifentanil infusion was stopped and muscle relaxation antagonism was performed after the skin was sutured. As the patient awakened and the mouth could open as directed, the SaCoVLM™ was removed. The patient was transferred to the Post Anesthesia Care Unit (PACU).

**Fig. 2:**
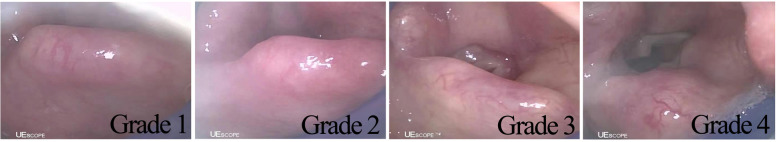
SaCoVLM™ Glottic exposure grades. Grade 1: visualization of the lateral part of the right aryepiglottic fold and part of the laryngeal inlet, and the ventilation was good; Grade 2: visualization of the bilateral aryepiglottic fold and part of laryngeal inlet, and the ventilation was good; Grade 3: visualization of all laryngeal inlet and posterior glottis; Grade 4: visualization of the whole glottis

We divided the glottic exposure into four grades under SaCoVLM™ (Fig. 2). Grade 1: visualization of the lateral part of the right aryepiglottic fold and part of the laryngeal inlet, and the ventilation was good; Grade 2: visualization of the bilateral aryepiglottic fold and part of laryngeal inlet, and the ventilation was good; Grade 3: visualization of all laryngeal inlet and partial glottis; Grade 4: visualization of the whole glottis. Fibreoptic bronchoscopy grade is as follows [7, 8]: Grade 1: visualization of no glottis; Grade 2: visualization of glottis and the lingual surface of epiglottis; Grade 3: visualization glottis and the laryngeal surface of the epiglottis. Grade 4:visualization of glottis.

### Data collection

The primary variables: visualization and grades of glottic exposure under SaCoVLM™. The secondary variables: the success rate of insertion, including the first-time success rate and the total success rate, the sealing pressure, classification under fibreoptic bronchoscopy, the insertion time, adjustment times. Other variables: intraoperative findings, secretions after SaCoVLM™ removal, soft tissue injuries (blood or bleeding), complications within postoperative 24 h (sore throat, hoarseness, difficulty swallowing).

### Statistical analysis

SPSS26.0 statistical software was used. We used means and standard deviation to describe continuous data and percentages for categorical data.

## Results

A total of 100 patients were recruited in this study, including 19 males (19%) and 81 females (81%), with an average age of 50.8 years, an average height of 164.1 cm and an average weight of 64.4 kg. The Demographic data are shown in Table 1.

**Table 1:** Demographic data

Variables	$\overline{\mathrm{\nu}}$ ± s/%
Gender	
Male (rate)	19 (19%)
Female (rate)	81 (81%)
Age	50.8 ± 12.2
Height (cm)	164.1 ± 7.3
Weight (kg)	64.4 ± 10.4
BMI (kg/m2)	23.9 ± 3.0
ASA	
I	62 (62%)
II	38 (38%)
Type of operation	
Gynecological surgery	68 (68%)
General surgery	26 (26%)
Urinary surgery	6 (6%)
Pneumoperitoneum	
Yes	62 (62%)
No	38 (38%)

*ASA* American Society of Anesthesiologists, *BMI* body mass index

The glottis exposure classification is showed in Table 2. All patients were observed under fiberoptic bronchoscopy to classify the glottis again.

**Table 2:** Glottis exposure classification

Classification	Grade 1	Grade 2	Grade 3	Grade 4
SaCoVLM™ (cases)	55 (55%)	23 (23%)	14 (14%)	8 (8%)
Fibreoptic bronchoscopy (cases)	1 (1%)	10 (10%)	14 (14%)	71 (71%)
Adjustment of SaCoVLM™ (cases)	0 (0%)	17 (17%)	33 (33%)	50 (50%)

SaCoVLM™ classification is obtained by camera observation through the SaCoVLM™ visual channel

Grade 1: visualization of the lateral part of the right aryepiglottic fold and part of the laryngeal inlet, and the ventilation was good

Grade 2: visualization of the bilateral aryepiglottic fold and part of laryngeal inlet, and the ventilation was good

Grade 3: visualization of all laryngeal inlet and posterior glottis

Grade 4: visualization of the whole glottis

Fibreoptic bronchoscopy classification is obtained by placing the Fibreoptic bronchoscopy in the distal opening of the SaCoVLM™ vent

Grade 1: visualization of no glottis

Grade 2: visualization of glottis and the lingual surface of epiglottis;

Grade 3: visualization glottis and the laryngeal surface of the epiglottis

Grade 4:visualization of glottis

The first-time success rate of SaCoVLM™ insertion was 95%(95% CI = 0.887 to 0.984), and the total success rate was 96%(95% CI = 0.901 to 0.989). One patient was successfully ventilated after size 4 was changed with size 3, three patients were intubated because of positive air leakage, and one patient was intubated because of excessive airway pressure. All the five patients underwent adjustments, including the up-down maneuver, raising jaw with both hands, the Chandy maneuver, increasing and reducing the amount of air, re-insertion and changing the laryngeal mask size. The average insertion time was 16.3 s. In all patients with good ventilation, the gastric tube was easy to insert. The average sealing pressure was 34.1 cmH_2_O, and 72% of SaCoVLM™s achieved a sealing pressure exceeding 30 cmH_2_O (Table 3).

**Table 3:** Outcomes of SaCoVLM™ insertion

Result	Cases (%)
The first-time success rate	95 (95%)(95% CI = 0.887 to 0.984)
The total success rate	96 (96%)(95% CI = 0.901 to 0.989)
SaCoVLM™ size	
size3	5 (5%)
size4	69 (69%)
size5	26 (26%)
Insertion adjustments	
Once	95 (96%)
Twice	5 (5%)
Insertion time(s)	16.3 ± 4.8
Ventilation	
Satisfaction	97 (97%)
Unable to ventilation	3 (3%)
Air leakage	
positive	3 (3%)
Negative	97 (97%)
Gastric intubation	
Positive	96 (96%)
Negative	1 (1%)
Sealing pressure (cmH_2_O)	34.1 ± 6.2
Peak airway pressure (cmH_2_O)	13.8 ± 3.0

Four cases of unsuccessful SaCoVLM™ were excluded and related complications were not followed up. During the study no gastric reflux or contraposition occurred in the 96 cases that were included in the final analysis. After removal, 7% of the SaCoVLM™s were stained with blood. One case had bleeding in the mouth, and 24% had secretions near the mask sac. The incidence of postoperative sore throat was 13%, without dysphagia and hoarseness (Table 4).

**Table 4:** Postoperative complications

Outcomes	$\overline{\mathrm{\nu}}$ ± s/%
Gastric drainage (ml)	9.5 ± 8.5
Cuff deflating volume (ml)	31.9 ± 7.2
Blood stains	
Yes	7 (7%)
No	89 (93%)
Active bleeding in oral cavity	
Yes	1 (1%)
No	95 (99%)
Secretions	
No	73 (76%)
Yes	23 (24%)
Postoperative sore throat	
Grade 0 (none)	83 (86%)
Grade 1 (slight)	12 (12%)
Grade 3 (medium)	1 (1%)
Dysphagia	
Yes	0 (0%)
No	96 (100%)
Hoarseness	
Yes	0 (0%)
No	96 (100%)

## Discussion

This SaCoVLM™ contains a camera to capture the images of the glottis and display them on a screen. We graded the images of 100 patients. Totaltrack mask can only visualize the glottis in 83% of patients [9]. LMA Ctrach, another video laryngeal mask, can expose the glottis in 85% of patients after manual adjustment [10]. Compared with the above two masks, SaCoVLM™ only visualized the glottis in eight cases after the first insertion, but in 83% patients after manual adjustment.. Only eight cases of glottis could be seen after SaCoVLM™ was placed for the first time. This was due to the insertion of the laryngeal mask using a blind insertion technique, resulting in too deep insertion of the laryngeal mask. Furthermore, the first-time success rate of SaCoVLM™ insertion was 95%, which was much higher than that of currently used LMA Supreme mask (77–88%) [11–14].

Alignment and sealing pressure are key factors that determine the effectiveness of a laryngeal mask airway. Sealing pressure can be used to identify the success of positive pressure ventilation, but also measure the airway protection [6]. We found that, after SaCoVLM™ insertion, only one case showed glottic exposure in grade 1 under fiberoptic bronchoscopy. The reason was that the epiglottis of this patient was large enough to cover the glottis. And 71 cases showed glottic exposure in grade 4, which indicated that this laryngeal mask could achieve good alignment. Under SaCoVLM™, all the 100 patients could display their partial or whole laryngeal inlet, which lays a foundation for the in-depth study of tracheal intubation. SaCoVLM™ achieved an average sealing pressure of 34.1cmH_2_O, over 30cmH_2_O in 72% of patients, both much higher than those achieved by the LMA Supreme mask [15–19]. Therefore, SaCoVLM™ can serve as an effective supraglottic airway management tool.

SaCoVLM™ can monitor the conditions of and surrounding the glottis during the whole operation. Because the patients are routinely fasted and forbidden to drink before operation, gastrointestinal drainage tubes are routinely placed. With SaCoVLM™, the drainage was smooth. Muscle relaxants were added on time and body position did not changed. Therefore, we found no regurgitation of stomach contents to the larynx and glottis-SaCoVLM™ disalignment during the operation. Related complications included sore throat (13%)(slight 12%, medium 1%) [20], blood staining on SaCoVLM™ (7%), and bleeding (1%), all of which were relieved 24 h after the operation. No serious complications, such as hoarseness or dysphagia, occurred. It was found that 19.6% of the patients developed sore throat in 24 h after the use of LMA Supreme (Laryngeal mask airway supreme) and 10% showed blood stains on the laryngeal mask [18]. Other studies showed that no blood stains occurred after removing LMA Supreme, and the incidence of sore throat was 70.6% [21]. In two studies using LMA Supreme, 7% ~ 10% of the patients presented blood stains after mask removal and 7% ~ 11.8% developed mild sore throat within 1 h after surgery [18, 22, 23]. Therefore, the incidence of complications related to SaCoVLM™ is similar to that of LMA Supreme, suggesting that it can be used safely in clinical practice.

In this study, we were unable to safely and effectively ventilate four patients with the SaCoVLM™. The airway was changed to an endotracheal tube after unsuccessful manual adjustment of the SaCoVLM™. The reasons were as follows: (1) After SaCoVLM™ insertion, the airway pressure was high to 32 cmH_2_O and the perilaryngeal soft tissue obstructed ventilation; (2) Air leakage test was positive after SaCoVLM™ insertion, and the epiglottis was still reflexed after manual adjustment; (3) Air leakage test was positive after SaCoVLM™ insertion, and the patient’s larynx was too high and the sealing effect of the mask was poor; (4) Size 5 was too small to match the patient who was 178 cm and 73 kg and had a large oral cavity.

In this study, the classification of SaCoVLM™ and fiberoptic bronchoscopy is quite different. The reason for the large difference is that they have different observation sites. FOB is through the laryngeal mask vent tube, and the glottis is observed at the open end of the vent tube. The SaCoVLM™ camera is located on the right side of vent cuff, which is equivalent to the right side of the vent opening end. The actual distance between the two observation sites is very small, about 0.5 cm. Although there is a great difference in the classification of glottis, there is no great difference in the actual alignment of laryngeal mask. The Table 2 shows that when the glottis can be well observed by SaCoVLM™, the alignment between LMA and glottis is very good. At the same time, it also lays a foundation for later intubation research.

Limitations also exist in this study. At present, the TotalTrack™ and LMA Ctrach™ are both video laryngeal masks, which can be used to visually guide endotracheal intubation when necessary [5, 24]. The SaCoVLM™ in this study also has the same function,but Totaltrack™ and LMA CTrach™ are not being widely used in China. Therefore, this study lacks a comparison of SaCoVLM™ with Totaltrack™ and LMA CTrach™. Second, we have mainly studied patients with Mallampati classes I-II, and this study is single center study with a relatively small sample size, therefore we are unable to determine the true effectiveness and safety of the SaCoVLM™. The reason for the small sample size is that the original purpose of this study is to be a preliminary study with a large multi-center sample size, so the sample size is small. Meanwhile all SaCoVLM™ insertions were performed by a single operator. This has the advantage of avoiding artificial errors in glottic exposure classification and leak pressure measurements, but may lead to limitations, such as a higher success rate for SaCoVLM™ than other laryngeal masks. Finally, only 19 males were included in the study population predominantly due to most surgeries being performed in gynaecological practice. This is also a limitation of this study. However, it has been pointed out in the literature that the selection of the laryngeal mask based on weight is equally effective as that based on gender [25]. In this study, the selection was based on weight, so the gender ratio was not strictly emphasized. This observational study as a simple initial study, aiming to lay a foundation for the later multi-center large-sample study. Although the results of the preliminary study look promising, further work is required to determine whether the LMA is effective and safe for use. Multi-center and larger-sample studies involving more Mallampati III-IV and male patients are needed to verify the effectiveness and safety of SaCoVLM™.

## Conclusions

In conclusion, SaCoVLM™ can visualize partial or whole laryngeal inlets during the surgery, with a high success rate, a high sealing pressure and smooth gastroesophageal drainage. SaCoVLM™ could be a promising new effective supraglottic device for airway management during general anesthesia.

## Data Availability

The datasets used and/or analyzed during the current study are available from the corresponding author on reasonable request.

## Abbreviations

SaCoVLM™: SaCoVLM™ video laryngeal mask
ECG: Electrocardiogram
HR: Heart rate
BP: Blood pressure
Sp0_2_: Oxygen saturation
BIS: bispectral index
PETCO2: End-tidal carbon dioxide
ASA: American Society of Anesthesiologists
BMI: Body mass index
MAP: Mean arterial pressure
LMA: Laryngeal mask airway
